# Early Changes in Resting-State Connectivity of the Anterior Insular Cortex Are Associated with Reductions in Pain and Catastrophizing After Total Hip Arthroplasty in Female Patients: A Preliminary Study

**DOI:** 10.3390/jcm15103799

**Published:** 2026-05-14

**Authors:** Yuji Chuda, Tsubasa Mitsutake, Atsushi Kawaguchi, Takanori Taniguchi, Hisato Nakazono, Mitsunori Okita, Maiko Sakamoto

**Affiliations:** 1 Department of Rehabilitation, Shiroishi Kyouritsu Hospital, Saga 849-1112, Japan; y.c.63415@gmail.com; 2 Graduate School of Medicine, Saga University, Saga 849-8501, Japan; 3 Education and Research Center for Community Medicine, Faculty of Medicine, Saga University, Saga 849-8501, Japan; mitsuba@cc.saga-u.ac.jp (T.M.); akawa@cc.saga-u.ac.jp (A.K.); 4 Department of Physical Therapy, Faculty of Medical Science, Fukuoka International University of Health and Welfare, Fukuoka 814-0001, Japan; taniguchi@takagigakuen.ac.jp; 5 Department of Occupational Therapy, Faculty of Medical Science, Fukuoka International University of Health and Welfare, Fukuoka 814-0001, Japan; nakazono@takagigakuen.ac.jp; 6 Department of Clinical Services, Shiroishi Kyouritsu Hospital, Saga 849-1112, Japan; seibindo@po.saganet.ne.jp

**Keywords:** arthroplasty, catastrophization, functional, hip, magnetic resonance imaging, neural pathways, neuronal plasticity, replacement

## Abstract

**Background/Objectives**: Chronic pain in osteoarthritis alters large-scale brain networks, including the insular cortex and default mode network. While total hip arthroplasty (THA) provides substantial relief, the early postoperative reorganization of functional connectivity (FC) remains unclear. This longitudinal fMRI study exploratively investigated how early improvements in pain intensity and catastrophizing are associated with insular FC alterations following THA. **Methods**: In this exploratory, longitudinal observational study, 10 female patients with hip osteoarthritis underwent resting-state fMRI and clinical assessments—Pain Visual Analogue Scale (VAS), Pain Catastrophizing Scale (PCS), and Japanese Orthopaedic Association (JOA) hip score—preoperatively and two weeks post-THA Whole-brain seed-to-voxel FC analyses were conducted using the bilateral anterior insular cortex as the seed. Changes in FC (ΔFC) were correlated with preoperative scores and postoperative clinical changes (ΔVAS, ΔPCS). **Results**: Following THA, VAS and PCS scores decreased significantly, while JOA scores improved. rs-fMRI analysis revealed that FC between the left anterior insula and major DMN regions as well as the right anterior cingulate cortex (ACC) increased significantly overall. Correlation analysis showed that greater reductions in pain intensity (ΔVAS) were significantly associated with increased ΔFC across these regions. Conversely, greater reductions in pain catastrophizing (ΔPCS) were associated with a suppression of these FC increases. **Conclusions**: Given the preliminary nature of this study, these findings suggest that the alleviation of pain catastrophizing following THA may be associated with the initial reorganization of the aIC network, rather than establishing a definitive causal relationship. Further large-scale longitudinal studies are required to confirm these potential neural signatures.

## 1. Introduction

Hip osteoarthritis is a progressive degenerative disorder characterized by articular cartilage breakdown, resulting in persistent pain and functional limitation. This disorder is particularly prevalent in women, and large-scale epidemiological studies in Japan have shown that women account for the majority of patients requiring surgical intervention for end-stage disease [1]. Total hip arthroplasty (THA) is widely recognized as the most effective surgical intervention for end-stage hip osteoarthritis, providing substantial pain relief and restoration of joint function [2]. Following surgery, most patients demonstrate marked improvements in gait and activities of daily living, leading to improved quality of life (QoL) [3]. Nevertheless, approximately 7–15% of patients report persistent pain, discomfort, or psychological distress despite successful implant positioning on imaging and objectively restored physical function [4,5]. This mismatch between clinical indicators and subjective outcomes suggests that hip osteoarthritis involves mechanisms beyond localized joint pathology [6].

Accumulating evidence from neuroscience indicates that chronic pain reflects not only persistent nociceptive input from peripheral tissues but also functional and structural alterations within the central nervous system [7,8,9]. Prolonged pain input induces central sensitization, a hypersensitive state that disrupts normal pain processing networks [10]. In particular, the insular cortex and anterior cingulate cortex (ACC), key components of the salience network involved in emotional and sensory pain processing, show hyperactivity and gray matter volume alterations in chronic pain states [11]. For instance, in patients with knee osteoarthritis, abnormal increases in functional connectivity (FC) between the anterior insula Cortex (aIC) and pain expectancy-related regions, such as the orbitofrontal cortex, have been reported [12], suggesting that insula-centered network abnormalities contribute to pain chronicity and severity in degenerative conditions [12].

Resting-state functional magnetic resonance imaging (rs-fMRI) enables noninvasive and objective assessment of these brain network alterations [13]. By analyzing correlations in low-frequency blood oxygen level-dependent (BOLD) signal fluctuations during rest, rs-fMRI quantifies FC without external task demands. This approach captures patient-specific baseline brain network organization and has been proposed as an objective biomarker of chronic pain [14,15]. A critical feature of chronic pain is the relationship between the aIC and the default mode network (DMN). The DMN, comprising the medial prefrontal cortex, posterior cingulate cortex (PCC), precuneus, and hippocampus, supports self-referential processing, memory, and emotional regulation during rest [16]. Normally, DMN activity is suppressed by external nociceptive input; however, in patients with chronic pain, this regulatory mechanism is disrupted, resulting in persistent pain-focused attention and abnormal insula–DMN connectivity [17,18]. Pain catastrophizing, characterized by exaggerated negative appraisal of pain, is strongly associated with enhanced coupling between the DMN and pain-related regions [19]. It should be noted, however, that the relationship between pain processing and DMN activity is highly complex and multifaceted, involving dynamic and bidirectional interactions rather than a simple on-off mechanism.

While chronic pain gradually reorganizes the DMN, the sudden withdrawal of peripheral nociceptive input following Total Hip Arthroplasty (THA) triggers a neural reorganization. However, the specific mechanisms that govern the decoupling of these established, abnormal CNS patterns remain poorly understood [20]. It is unclear whether pain relief alone results in immediate reorganization of brain networks or whether cognitive and emotional factors, including pain catastrophizing, differentially influence functional recovery. Resolving this issue is essential for optimizing postoperative rehabilitation strategies.

This study aimed to explore how changes in pain intensity, representing the sensory dimension, and pain catastrophizing, representing the cognitive dimension, are differentially associated with reorganization of aIC-centered brain FC following THA. We hypothesized that reduced postoperative pain would modulate hyperactive insula–DMN connectivity, whereas pain catastrophizing would exert a distinct influence on brain network plasticity, reflecting a unique bias within the neural networks. Given the exploratory nature and small sample size of this pilot study, our investigation focuses on identifying potential neural signatures rather than establishing definitive causal relationships.

## 2. Materials and Methods

### 2.1. Participants

This study was designed as an exploratory, longitudinal observational study. Due to its preliminary nature as a pilot study, it was not prospectively registered on a clinical trial platform. Patients scheduled for THA at Shiroishi Kyouritsu Hospital (Saga, Japan) were recruited using consecutive sampling. The inclusion criteria required participants to be right-handed; no restrictions were placed on gender. Exclusion criteria were (1) regular use of centrally acting medications (antidepressants, anxiolytics, antiepileptics, opioids, etc.); (2) history of neurological or psychiatric disorders or head trauma; (3) dementia or cognitive impairment; (4) chronic pain requiring treatment outside the hip joint (e.g., rheumatoid arthritis, fibromyalgia); and (5) contraindications to MRI (e.g., claustrophobia, metal implants). Use of non-steroidal anti-inflammatory drugs (NSAIDs) and acetaminophen for standard perioperative pain management was permitted. Postoperative rehabilitation followed the hospital’s standard program, with activities adjusted according to the patient’s pain tolerance. The program consisted of two 20- to 40-min sessions per day (morning and afternoon), including range-of-motion exercises, muscle strengthening, and gait training; specialized psychological interventions such as cognitive behavioral therapy or pain neuroscience education (PNE) were not included. The study was conducted in accordance with the Declaration of Helsinki and approved by the Ethics Committee of Shiroishi Kyouritsu Hospital (Approval No. 20266). Written informed consent was obtained from all patients.

### 2.2. Measurement Procedure

Assessments were conducted at two time points: preoperatively on the day before surgery and postoperatively at 2 weeks (day 14). At each time point, patients underwent fMRI scanning and clinical evaluation.

### 2.3. Clinical Assessment

Pain intensity was assessed using the Visual Analogue Scale (VAS), evaluating maximum pain during movement, including walking and rising. The VAS is a reliable and valid instrument widely used in clinical trials [21]. Pain catastrophizing was measured using the Pain Catastrophizing Scale (PCS), which comprises three dimensions: rumination, magnification, and helplessness. The PCS has demonstrated predictive validity for pain outcomes, and the Japanese version has high internal consistency and criterion-related validity [22]. In addition, the Japanese Orthopaedic Association (JOA) hip score was used as an objective assessment of hip function, for which reliability and validity have been established [23]. Furthermore, the usage of analgesics (NSAIDs, acetaminophen, or their combination) was recorded. Clinical assessments were performed preoperatively and at 2 weeks postoperatively, and both absolute values and changes (Δ = Post − Pre) were calculated for each scale. In this calculation, a negative value for the change in clinical scores indicates a reduction in symptoms.

### 2.4. MRI Acquisition

MRI scans were acquired using a 1.5 T scanner (Nova Dual, Philips, Best, The Netherlands) to obtain resting-state functional magnetic resonance imaging (rs-fMRI). Participants were instructed to keep their eyes closed, remain awake, and avoid focused thoughts. Wakefulness was verbally confirmed after scanning. Functional images were acquired with Gradient Echo-Planar Imaging using the following parameters: repetition time (TR) = 2985 ms, echo time (TE) = 50 ms, flip angle (FA) = 90°, field of view (FOV) = 230 × 230 mm, matrix = 128 × 128, voxel size = 1.80 × 1.80 × 4.00 mm, slice thickness = 4 mm (33 axial slices), resulting in a total of 80 volumes. High-resolution three-dimensional T1-weighted images were acquired for spatial normalization (TR = 7.98 ms, TE = 3.7 ms, FA = 9°, FOV = 230 × 230 mm, matrix = 576 × 576, voxel size = 0.4 × 0.4 × 1.2 mm, slice thickness = 1.2 mm, 270 axial slices, inversion time = 940 ms).

### 2.5. fMRI Data Preprocessing

Preprocessing was conducted using the CONN Toolbox (version 22.v2407; The Gabrieli Lab, Massachusetts Institute of Technology, Cambridge, MA, USA) [24] in MATLAB R2024b (MathWorks, Inc., Natick, MA, USA) with SPM25 (version 25.01.02; Statistical Parametric Mapping; Wellcome Centre for Human Neuroimaging, University College London, London, UK) as the underlying software [25]. Functional images underwent head motion and slice-timing correction, followed by co-registration with each participant’s T1 structural image and normalization to the Montreal Neurological Institute space. Spatial smoothing was applied using a Gaussian kernel with full width at half maximum of 8 mm. Physiological and non-neural noise was minimized using the anatomical component-based noise correction method (aCompCor) [26]. Notably, global signal regression (GSR) was not applied, in accordance with the standard aCompCor pipeline. Principal components from white matter and cerebrospinal fluid, head motion parameters (six translation/rotation components and their first derivatives), and linear trends were regressed out. A band-pass filter of 0.008–0.09 Hz was applied to isolate low-frequency fluctuations. Framewise Displacement (FD) [27] was calculated and included as a covariate in second-level analyses to control for head motion artifacts and signal-to-noise (S/N) limitations inherent in 1.5 T acquisition. The mean FD across all participants was 0.073 ± 0.026 mm, confirming that head motion was well controlled. No participants were excluded for excessive motion (mean FD > 0.3 mm) [28].

### 2.6. fMRI Analysis

Bilateral anterior insular cortices were defined as regions of interest (ROIs) based on the Harvard–Oxford Cortical Structural Atlas (Center for Morphometric Analysis, Massachusetts General Hospital, Charlestown, MA, USA) [29] implemented in the CONN Toolbox. The aIC plays a central role in the pathophysiology of chronic pain and neuroplastic changes following treatment [30,31]. In particular, longitudinal studies have demonstrated that pain relief and improvements in psychological factors before and after joint replacement surgery are associated with changes in the functional connectivity of brain networks involving the aIC [32]. To examine overall FC within the Salience Network, which is involved in pain processing, these ROIs were used as seed regions. For each patient, Seed-to-Voxel analysis calculated Pearson correlation coefficients between the mean BOLD signal of each seed and the time-series signal of every voxel in the brain. Correlation coefficients were transformed using Fisher’s *r*-to-*z* to approximate a normal distribution. At the group level, changes in FC (ΔFC = Post − Pre) were evaluated with paired *t*-tests. In this analysis, the aforementioned mean framewise displacement (FD) was included as a covariate in the model to statistically control for the effects of head motion. To balance the risk of Type I and Type II errors in this preliminary investigation, statistical significance was set at a voxel-level threshold of *p* < 0.001 (uncorrected), combined with a cluster-level false discovery rate (FDR) correction of *p* < 0.05. This approach is widely utilized in exploratory neuroimaging studies to maintain sensitivity to early neural changes while ensuring a rigorous height threshold. Significant clusters were visualized and figures generated using MRIcroGL (version 1.2.20220720c; Chris Rorden, University of South Carolina, Columbia, SC, USA) [33].

### 2.7. Statistical Analysis

The Wilcoxon signed-rank test was used to compare clinical measures preoperatively and at 2 weeks postoperatively, and McNemar’s test was used to compare the proportions of medication use. For correlation with clinical measures, clusters significant at the group level (cluster-level FDR *p* < 0.05) were selected. ΔFC for each cluster was calculated as the difference between postoperative and preoperative *Z*-scores (Fisher *r*-to-*z* transformed). Considering the sample size, Spearman’s rank correlation coefficient (*ρ*) assessed relationships between ΔFC and both preoperative scores (Pre-Pain, Pre-PCS) and changes in clinical scores (ΔPain, ΔPCS; all calculated as Post − Pre). Analyses were performed using R (version 4.5.1; R Foundation for Statistical Computing, Vienna, Austria), with significance defined as *p* < 0.05. Scatter plots were used to visually confirm the absence of outliers prior to analysis. Due to the limited sample size, the total PCS score, rather than individual subscale scores, was used for functional connectivity analyses to minimize the risk of false positives. Furthermore, to evaluate the variability and uncertainty of the results in a small sample size, a bootstrap method with 2000 resamples was performed to calculate the 95% confidence intervals (CIs) for the correlation coefficients.

## 3. Results

### 3.1. Clinical Outcomes

A total of 10 patients scheduled for THA during the study period were screened; all 10 met the inclusion criteria, and no patients were excluded. There were no dropouts during the 2-week follow-up period, and all 10 participants completed the protocol and were included in the final analysis. Among them, four undergoing right hip and six undergoing left hip arthroplasty. Reflecting the epidemiological characteristics of hip osteoarthritis, all participants were female. At 2 weeks postoperatively, both Pain VAS and PCS scores decreased significantly, while JOA scores increased significantly, compared with preoperative values (*p* < 0.05; Table 1). No significant differences were observed in the use of perioperative analgesics (NSAIDs and acetaminophen) between pre- and postoperative periods (Table 1).

### 3.2. Resting-State FC Changes

**Figure 1:**
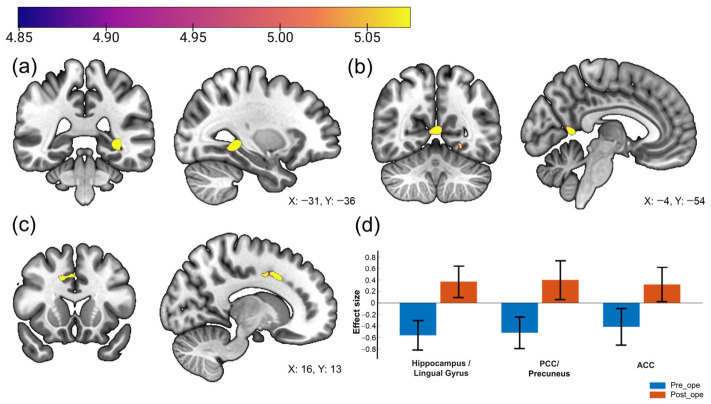
Significant changes in functional connectivity with the left insula seed. (**a**–**c**) Statistical parametric maps showing significant clusters in the (**a**) left hippocampus/lingual gyrus, (**b**) posterior cingulate cortex (PCC)/precuneus, and (**c**) right anterior cingulate cortex (ACC). The color bar represents *t*-values, with yellow indicating higher statistical significance. (**d**) Bar plots indicating the mean effect sizes (Fisher’s *z*-scores) for each cluster. Blue bars represent pre-operative values, and orange bars represent post-operative values. Error bars indicate standard error of the mean. Note: Results were thresholded at cluster-level *p* < 0.05 (FDR-corrected) with a voxel-level height threshold of *p* < 0.001.

Whole-brain voxel-wise Seed-to-Voxel analysis identified significant postoperative increases in FC between the left anterior insular cortex and the left hippocampus/lingual gyrus, PCC/precuneus, and right ACC (cluster-level FDR corrected *p* < 0.05; Figure 1, Table 2). Analyses using the right anterior insular cortex as a seed revealed no regions with significant FC changes. No regions exhibited significantly decreased connectivity with the left anterior insular cortex postoperatively.

### 3.3. Correlations Between Clinical Scores and FC

**Figure 2:**
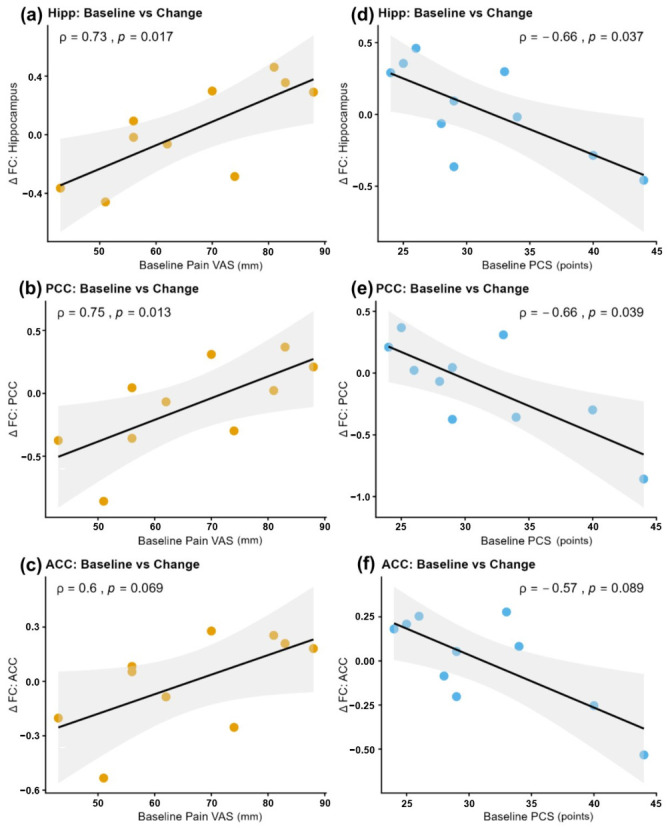
Association between preoperative clinical scores and postoperative changes in functional connectivity. Scatter plots showing the relationships between preoperative clinical scores (*x*-axis) and changes in functional connectivity (ΔFC; Post − Pre) (*y*-axis). Orange and blue plots represent the correlations with preoperative VAS and PCS scores, respectively. (**a**–**c**) A significant positive correlation was observed between preoperative VAS scores and ΔFC in the Hippocampus, PCC, and ACC, indicating that higher baseline pain was associated with greater increases in FC. (**d**–**f**) A significant negative correlation was observed between preoperative PCS scores and ΔFC in the same regions, indicating that higher baseline pain catastrophizing was associated with suppressed increases or decreases in FC. Values indicate Spearman’s rank correlation coefficients (*ρ*) and *p*-values. Shaded areas represent 95% confidence intervals calculated using the bootstrap method. Abbreviations: VAS = Visual Analogue Scale; PCS = Pain Catastrophizing Scale; FC = functional connectivity; Hipp = left Hippocampus; PCC = left Posterior Cingulate Cortex; ACC = right Anterior Cingulate Cortex.

Correlation analyses using the extracted ROI data revealed significant associations between preoperative clinical scores and postoperative changes in FC. In the hippocampus, higher preoperative pain intensity correlated with greater postoperative FC increases (*ρ* = 0.73, *p* = 0.017), whereas higher preoperative PCS scores were associated with smaller FC increases (*ρ* = −0.66, *p* = 0.037). A similar pattern was observed in the PCC, with preoperative pain positively correlating with FC increase (*ρ* = 0.75, *p* = 0.013) and preoperative PCS negatively correlating (*ρ* = −0.66, *p* = 0.039). In the ACC, trends aligned with other regions, although statistical significance was not reached (Pain: *ρ* = 0.60, *p* = 0.069; PCS: *ρ* = −0.57, *p* = 0.089; Figure 2, Table 3). All major regions showed correlation coefficients exceeding 0.6, indicating strong associations.

**Figure 3:**
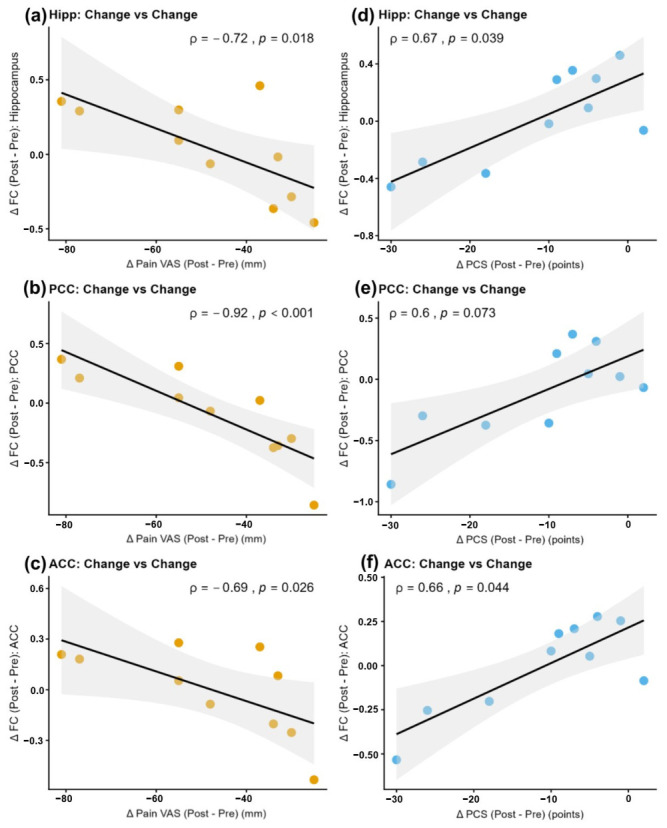
Correlations between changes in clinical symptoms and functional connectivity. Scatter plots illustrating the associations between changes in clinical scores (ΔClinical; Post − Pre) and changes in functional connectivity (ΔFC). Orange and blue plots represent the correlations with ΔVAS and ΔPCS, respectively. (**a**–**c**) A significant negative correlation was observed between ΔVAS and ΔFC in the Hippocampus, PCC, and ACC, indicating that a greater decrease in pain (negative ΔVAS) was associated with increased FC. (**d**–**f**) A significant positive correlation was observed between ΔPCS and ΔFC in the same regions, indicating that a greater decrease in pain catastrophizing (negative ΔPCS) was associated with suppressed increases in FC. Values indicate Spearman’s rank correlation coefficients (*ρ*) and *p*-values. Shaded areas represent 95% confidence intervals calculated using the bootstrap method. Note that negative values on the *x*-axis indicate clinical improvement (reduction in symptoms). Abbreviations: VAS = Visual Analogue Scale; PCS = Pain Catastrophizing Scale; FC = functional connectivity; Hipp = left Hippocampus; PCC = left Posterior Cingulate Cortex; ACC = right Anterior Cingulate Cortex.

Examining the relationship between changes in clinical symptoms (ΔVAS and ΔPCS) and ΔFC revealed contrasting patterns for pain and pain catastrophizing. Greater reductions in VAS scores were significantly associated with increased FC in the hippocampus (*ρ* = −0.72, *p* = 0.018), PCC (*ρ* = −0.92, *p* < 0.001), and ACC (*ρ* = −0.69, *p* = 0.026). Conversely, greater reductions in PCS scores correlated with significantly suppressed increases in FC in the hippocampus (*ρ* = 0.67, *p* = 0.039) and ACC (*ρ* = 0.66, *p* = 0.044). A similar correlation trend was observed in the PCC, although not significant (*ρ* = 0.60, *p* = 0.073; Figure 3, Table 3). Similarly, strong correlations with |*ρ*| > 0.6 were confirmed in many regions here as well.

## 4. Discussion

This study evaluated post-operative improvements in pain and pain catastrophizing following THA. Specifically, we exploratively examined functional connectivity (FC) within the insular cortex to investigate whether neural reorganization occurs as early as two weeks postoperatively. Although the sample size is limited, this preliminary study may capture the initial stages of essential neural mechanisms underlying the early recovery process from chronic pain.

The most significant and novel preliminary finding is that pain relief and reductions in pain catastrophizing were not reflected as uniform FC changes but as divergent plasticity. Specifically, greater improvement in physical pain intensity (sensory aspect) was associated with strengthened connectivity between the left anterior insula and the DMN, whereas greater improvement in pain catastrophizing (cognitive/emotional aspect) was linked to attenuated or normalized connectivity in the same regions. In chronic pain treatment, the sensory and cognitive recovery mechanisms are rarely distinguished. Based on these results, we hypothesize that postoperative brain functional recovery may involve at least two interrelated processes: a potential network reintegration accompanying the stabilization of sensory input and attenuation of hyperactivity associated with the release of cognitive bias.

In patients with severe preoperative pain who experienced greater postoperative pain relief, FC between the left anterior insula and the DMN (specifically the left hippocampus, PCC, and right ACC) was significantly enhanced. This likely reflects reintegration of networks fragmented by chronic pain. Chronic nociceptive input continually consumes brain resources [34]. From the perspective of large-scale brain networks, it has been pointed out that hyperactivity in the Salience Network (including the anterior insula) suppresses DMN function, which should normally be active at rest, consequently forming the basis for cognitive impairments in self-referential processing, memory integration, and attentional switching [35]. The increased connectivity observed here likely indicates that pain relief provided by THA removed cognitive interference from nociception, supporting dynamic left anterior insula–DMN interactions. Increased right ACC connectivity may reflect recovery of emotional regulation rather than heightened pain processing, as the ACC encodes the unpleasant component of pain [36]. This suggests a release from the rigid network state caused by pain. Strengthened hippocampal connectivity is also notable, as chronic pain is associated with hippocampal dysfunction and atrophy [37], and abnormal hippocampal–DMN connectivity correlates with pain intensity in osteoarthritis [38]. We hypothesize that Postoperative enhancement could potentially reflect updating of memory and body representation from a prior pain-associated unpleasant body image to a new, pain-free body schema capable of moving without pain.

Conversely, patients with higher preoperative PCS scores who showed greater improvement in pain catastrophizing exhibited significantly suppressed increases in connectivity between the left anterior insula and major DMN regions (left hippocampus, PCC) and the right ACC. This finding may initially seem contradictory to the reintegration described above; however, it aligns with the pathophysiology of pain catastrophizing, which involves excessive attention to pain and rumination on negative predictions. Neuropsychologically, this manifests as pathological hyperconnectivity between the anterior insula, processing interoception, and the DMN (PCC for self-referential processing, ACC for emotional evaluation) [18]. In the brain of patients with high PCS, minor sensory inputs (left anterior insula activity) were immediately misinterpreted as threats to the self (PCC/ACC activity), creating a persistent loop of anxiety. Therefore, the postoperative suppression of FC increases likely represents attenuation or normalization of this pathological loop, reflecting a reduction in fear of pain and anticipatory anxiety. These results indicate that weakening maladaptively strengthened circuits is as essential as strengthening specific network connections in chronic pain recovery [39].

In this study, the final cohort consisted exclusively of female patients. While this was a result of consecutive recruitment based on our clinical inclusion criteria, it provided a significant methodological advantage. Previous studies have indicated clear sex differences in pain sensitivity and resting-state functional connectivity patterns [40,41]. Therefore, in a small sample size of 10 participants, including both sexes could have introduced sex-based heterogeneity in brain function as noise, potentially confounding the results. Consistent with the epidemiological characteristics of the disease, focusing on a single-sex cohort maximized sample homogeneity, allowing for a more sensitive and valid detection of the specific FC changes associated with the surgery. However, it should be noted that these findings may not be directly generalizable to male patients, and further studies including both sexes are required to confirm the universality of these neural changes.

Another notable finding is that these complex network reorganizations occurred within only 2 weeks postoperatively. Recovery from gray matter reduction associated with chronic pain typically requires months to years [20], whereas the FC changes observed here likely represent a more immediate adaptive process preceding structural plasticity. Rapid changes may arise from increased transmission efficiency in existing synapses (long-term potentiation/depression) or disinhibition of suppressed pathways rather than new synapse formation [42]. Clinically, this suggests that the early postoperative period is critical for functional brain plasticity. The resolution of hip pain drastically alters peripheral input, triggering large-scale central nervous system reorganization, and the subjective experience of patients aligns with dynamic network changes. These findings highlight the importance of early rehabilitation, not only for physical training but also for adaptive brain relearning. Furthermore, the standard physical therapy provided during the early postoperative period likely contributed to improved physical function and self-efficacy, which may have influenced the modulation of pain catastrophizing and pain perception.

Regarding future research directions, exploring, a multifaceted approach that incorporates cognitive rehabilitation alongside conventional exercise therapy could be a valuable hypothesis. For instance, Pain Neuroscience Education (PNE), which facilitates accurate understanding of pain mechanisms and reduces unnecessary anxiety and catastrophizing, may hold potential [43]. Future clinical trials could investigate whether combining PNE with cognitive behavioral therapy, as needed [44,45], can further mitigate excessive anterior insula–DMN connectivity [46]. These neuroscientific findings support interpreting the presently observed suppression of aIC-DMN connectivity as a reorganization process of maladaptive neural circuits. We hypothesize that integrating such psychological approaches may alleviate the negative cognitive-emotional loop and promote adaptive network reorganization. Additionally, biofeedback interventions might be another avenue to explore, as they have been shown to normalize insular and anterior cingulate cortex activity and enhance pain control [47,48]. Since the current study observed significant neural reorganization even in the absence of specialized psychological interventions, it remains unclear whether these changes were driven solely by surgical pain relief or by standard physical therapy; identifying the specific drivers of the reduction in pain catastrophizing warrants further investigation through multifaceted interventions. Future adequately powered studies are needed to determine if integrating cognitive and emotional interventions into rehabilitation and routine care, rather than focusing solely on the affected joint, can improve long-term QoL by promoting adaptive brain network reorganization.

### Limitations and Future Directions

This study is subject to several significant limitations that warrant careful consideration, which can be categorized into three main areas. First, regarding study design and sampling, the sample size was small (10 participants), which inherently limits statistical power and increases the risk of Type I errors (false-positive results). Furthermore, although no restrictions were placed on gender during recruitment, the final cohort consisted exclusively of female patients with hip osteoarthritis. While this maximized sample homogeneity for this initial investigation, the findings may not be directly generalizable to the male population. Additionally, the absence of a control group (e.g., healthy individuals) prevents the definitive isolation of surgery-specific effects from confounding factors such as natural recovery. Second, several methodological and temporal constraints must be noted. Although assessments were conducted before and after surgery to evaluate changes, the postoperative follow-up was limited to two weeks. This duration is too short to determine the long-term stability of brain network reorganization or permanent recovery. At this early stage, the observed alterations might partially reflect acute postoperative physiological stress rather than permanent cortical reorganization. Methodologically, the use of a 1.5 T MRI scanner, rather than a higher-field 3 T system, resulted in a lower signal-to-noise ratio, potentially affecting the accuracy of the functional connectivity data. Moreover, although analgesic use (NSAIDs and acetaminophen) was permitted and monitored, its potential influence on neurovascular coupling and the BOLD signal cannot be entirely excluded. Third, concerning the scope and interpretation of the results, the exploratory nature of this study and its lack of prospective registration mean that causal relationships between neural changes and clinical improvements cannot be firmly established. Furthermore, while we demonstrated improvements in objective physical function (JOA scores), our analysis focused primarily on subjective psychological factors; future research should investigate the direct relationship between neural reorganization and objective functional recovery. Finally, the exclusion of specialized psychological interventions (e.g., cognitive behavioral therapy) limits the interpretation of the specific factors driving observed reductions in pain catastrophizing. Future large-scale, registered clinical trials utilizing 3 T MRI and longitudinal tracking (e.g., six months to one year) are essential to confirm these preliminary signatures.

## 5. Conclusions

This study demonstrated that drastic pain relief and reduced pain catastrophizing following THA are associated with divergent plasticity in anterior insula–DMN connectivity during the early postoperative period. Enhancement of connectivity corresponding to improved pain intensity reflects reintegration of networks disrupted by chronic pain, whereas the suppression of FC increases associated with reduced pain catastrophizing indicates attenuation of pathological hyperconnectivity. In conclusion, while definitive causal relationships cannot be established from this exploratory pilot study, our preliminary findings suggest that postoperative brain functional recovery following THA is not a uniform process. These changes may be associated with distinct neural signatures related to both sensory modulation and the reduction of cognitive bias. Clinically, these results may provide a neuroscientific basis for future studies to investigate multifaceted rehabilitation approaches for THA patients.

## Figures and Tables

**Table 1:** Characteristics of Participants and Clinical Outcomes (*N* = 10).

Characteristic	Preoperative	Postoperative (2 Weeks)	Effect Size (*r*)	*p*-Value
Age (years)	71.1 ± 11.8	-	-	-
Sex (Female)	10	-	-	-
Body Mass Index (kg/m^2^)	25.7 ± 7.2	-	-	-
Side of surgery (R/L)	4/6	-	-	-
Pain VAS (mm)	66.4 ± 15.1	18.9 ± 15.4	0.62	0.006 *
PCS (total score)	31.2 ± 6.6	20.4 ± 6.8	0.57	0.011 *
JOA hip score	46.4 ± 18.5	62.2 ± 14.5	0.50	0.025 *
Medication, *n* (%)				
NSAIDs	6 (60.0%)	7 (70.0%)	-	1.000 ^†^
Acetaminophen	6 (60.0%)	8 (80.0%)	-	0.500 ^†^
Combination	5 (50.0%)	5 (50.0%)	-	1.000 ^†^

Note: Values are mean ± SD or number. VAS = Visual Analogue Scale; PCS = Pain Catastrophizing Scale; JOA = Japanese Orthopaedic Association. * Significant difference between pre and postoperation (*p* < 0.05, Wilcoxon signed-rank test). *r* = effect size (*Z*/√*N*). ^†^ *p*-values were calculated using McNemar’s test (exact). NSAIDs: Non-steroidal anti-inflammatory drugs. Combination: Concomitant use of NSAIDs and Acetaminophen.

**Table 2:** Brain regions showing significant changes in functional connectivity with the left anterior insula seed.

Brain Region	Cluster Size (*k*)	Hemisphere	MNI Coordinates (x, y, z)	Peak *t*-Value	*p*-Value (FDR-Corr)
Hippocampus/ Lingual Gyrus	203	L	−32, −38, −02	10.85	0.003
Precuneus/PCC	102	L/Mid	−04, −54, +06	6.65	0.035
ACC	95	R	+16, +08, +42	11.41	0.035

Note: MNI = Montreal Neurological Institute; PCC = Posterior Cingulate Cortex; ACC = Anterior Cingulate Cortex; FDR = false discovery rate; L = Left; R = Right. Results were thresholded at cluster-level *p* < 0.05 (FDR-corrected) with a height threshold of *p* < 0.001 (uncorrected).

**Table 3:** Associations between changes in functional connectivity and clinical variables.

Brain Region (ΔFC)	Pain Intensity (VAS)		Pain Catastrophizing (PCS)	
	Baseline (Pre)	Change (Δ)	Baseline (Pre)	Change (Δ)
Hippocampus	0.73 [0.22, 0.92] (*p* = 0.017)	−0.72 [−0.99, −0.13] (*p* = 0.018)	−0.66 [−0.95, −0.11] (*p* = 0.037)	0.67 [−0.03, 0.99] (*p* = 0.039)
Posterior Cingulate Cortex	0.75 [0.23, 0.96] (*p* = 0.013)	−0.92 [−1.00, −0.61] (*p* < 0.001)	−0.66 [−0.96, −0.04] (*p* = 0.039)	0.60 [−0.25, 0.92] (*p* = 0.073)
Anterior Cingulate Cortex	0.60 [0.01, 0.88] (*p* = 0.069)	−0.69 [−1.00, −0.12] (*p* = 0.026)	−0.57 [−0.95, 0.15] (*p* = 0.089)	0.66 [−0.08, 1.00] (*p* = 0.044)

Note: FC = functional connectivity; VAS = Visual Analogue Scale; PCS = Pain Catastrophizing Scale; Δ = change score (postoperative minus preoperative). Values are Spearman’s rank correlation coefficients (*ρ*) with 95% confidence intervals [CI] in brackets and exact *p*-values in parentheses. CIs were calculated using a bootstrap method (2000 resamples).

## Data Availability

The data presented in this study are available on request from the corresponding author due to privacy restrictions.

## Abbreviations

The following abbreviations are used in this manuscript:

Abbreviation	Full Name
THA	Total Hip Arthroplasty
OA	Osteoarthritis
BMI	Body Mass Index
ADL	Activities of Daily Living
QoL	Quality of Life
NSAIDs	Non-Steroidal Anti-Inflammatory Drugs
rs-fMRI	Resting-State Functional Magnetic Resonance Imaging
BOLD	Blood Oxygenation Level Dependent
ROI	Region of Interest
MNI	Montreal Neurological Institute
SPM	Statistical Parametric Mapping
FD	Framewise Displacement
FC	Functional Connectivity
aIC	Anterior Insular Cortex
DMN	Default Mode Network
ACC	Anterior Cingulate Cortex
PCC	Posterior Cingulate Cortex
FDR	False Discovery Rate
VAS	Visual Analogue Scale
PCS	Pain Catastrophizing Scale
PNE	Pain Neuroscience Education
VBM	Voxel-Based Morphometry
